# Cross-Education Related to the Ipsilateral Limb Activity on Monopedal Postural Control of the Contralateral Limb: A Review

**DOI:** 10.3389/fphys.2020.00496

**Published:** 2020-05-21

**Authors:** Thierry Paillard

**Affiliations:** Laboratoire Mouvement, Equilibre, Performance et Santé, EA 4445, E2S/Université de Pau et des Pays de l’Adour, Département STAPS, Tarbes, France

**Keywords:** exercise, training, fatigue, motor skills, postural balance, balance, cross-education, cross-effects

## Abstract

Cross-education is the effect whereby the ipsilateral limb training generates contralateral effects as part of motor tasks requiring strength and skills. However, it is not yet known if cross-education applies to postural control which could be essential as part of human motricity. Hence, this review addresses the possible effects of acute and chronic unilateral exercises (i.e., fatiguing exercises and regularly repeated/training exercises, respectively) on the contralateral monopedal postural control. Evidence suggests that fatiguing exercises disturb the contralateral monopedal postural control. This disturbance emanates from spinal and supra-spinal alterations which provokes changes to the motor function of the contralateral limb and degrades its postural control. Unilateral training produces cross-education related to postural control, especially when it includes balance exercises, but this remains to be tested when it includes resistance exercises. Mechanistic explanations are proposed to explain how neurophysiological changes operate in the disturbance or improvement of the contralateral monopedal postural control after unilateral fatiguing exercises or training exercises (respectively) of the lower-limb.

## Introduction

Cross-education (also named cross-effects, cross-training effect, contralateral effect, cross-limb transfer, inter-limb transfer, bilateral transfer) is when motor output is improved in the untrained limb (i.e., the contralateral limb) after unilateral exercise training (i.e., in the ipsilateral limb). This can be observed in both healthy and pathological (young and older) subjects (Dragert and Zehr, 2011, 2013; Barss et al., 2016). The improvement of motor output only affects the homologous muscles in the contralateral limb and occurs during both training of muscle strength or learning of motor skills (Frazer et al., 2018). In general, an improvement of motor output (i.e., performance in muscle strength and/or motor skills) is the result of either structural adaptations (i.e., muscle hypertrophy) or functional adaptations (neurological adaptations) (Dankel et al., 2019). However, only real repetitive muscle contractions are likely to exert the mechanical stimuli necessary to trigger the process of synthesis of contractile proteins responsible for muscle hypertrophy and thus the improvement of muscle strength or motor output (Coffey and Hawley, 2007). Since ipsilateral muscle contractions do not involve any tangible contralateral muscle contractions [even if a certain level of tonic activity in the homologous muscles of the contralateral side can be observed (Devine et al., 1981)], muscle hypertrophy in the homologous muscles in the contralateral limb cannot be identified (Andrushko et al., 2018). Hence, cross-education can be characterized by a functional nervous modification in muscles activation. Thus, the improvement of motor output emanates rather from central adaptations (central nervous system) than peripheral adaptations (muscle tissue) (Andrushko et al., 2018). These adaptations concern both supraspinal (cortical and sub-cortical) and spinal levels (Frazer et al., 2018).

At the supraspinal level, neural adaptations caused by cross-education involve both structural and functional changes within cortical motor and non-motor regions (Frazer et al., 2018). Such changes are likely to subtly modify the functioning of the chain of motor command related to corticospinal excitability (increase), cortical inhibition (reduction), interhemispheric inhibition (reduction) as well as the voluntary activation associated to new regions of cortical activation (Frazer et al., 2018). In the cortical motor region, unilateral voluntary movement implies activation of the contralateral cortex in the same motor area via the corpus callosum (Kristeva et al., 1991). At the spinal level, the repetition of unilateral voluntary contractions (unilateral training) modifies the excitability of spinal motor pathways projecting to the contralateral side (Frazer et al., 2018). Presynaptic inhibition of Ia afferent motoneuron synapses would be partly at the origin changes in spinal networks and reciprocal inhibition (Hortobagyi et al., 2003). Reciprocal inhibition is mediated via specific interneurons i.e., Ia inhibitory interneurons located in the anterior horn which receive inputs from both segmental and supraspinal centers and which operate as an integration center in the spinal control of motricity (Lee and Carroll, 2007).

Whilst it is known that ipsilateral training can generate contralateral effects through motor tasks requiring strength and skills (Green and Gabriel, 2018), the possible contralateral effects related to the requirements for carrying out the movement, such as postural control, remains nevertheless to be established. Indeed, postural control turns out to be fundamental as part of human motricity (e.g., locomotion, motor action) specially in injured/pathological, frail and/or older, and sports subjects in terms of functional rehabilitation, prevention of falls, and sport performance optimization, respectively (Paillard, 2017).

The present work therefore aims to provide an overview of cross-education related to postural control – i.e., the study of effects of the ipsilateral limb activity on the contralateral monopedal postural control – and identify possible mechanistic explanations. In the context of unilateral orthopedic injuries, nerve trauma, stroke (or other unilateral pathologies) or unilateral functional frailties, this could enable physicians and sport trainers to improve their therapeutic strategies and (re)training strategies (after rehabilitation program), respectively, in order to enhance (or avoid losing) the monopedal postural abilities of the initially affected limb and limit its risk of new injury (e.g., from a fall) in patients and athletes.

## Factors Considered in Analyzing the Possible Cross-Education Phenomenon Related to Postural Control

The framework for the analysis of cross-education as well as postural control needs to be clarified.

### Analysis of Cross-Education

The analysis of the possible existence of cross-education related to the ipsilateral limb activity on monopedal postural control of the contralateral limb requires the evaluation of the effects of chronic/regular exercise (i.e., training effects). In order to refine this analysis it would also be appropriated to explore the effects of acute exercise since the chronic effects result from induced effects in each training exercise (i.e., the sum of exercise performed). The efficient training consists of carrying out exercise sessions sufficiently intense and/or long in order to induce beneficial physiological adaptations. Generally, this type of session generates muscle fatigue (particularly disturbing regarding postural control) which is reversible after a recovery period but constitutes a factor of fall risk for a while immediately after the session especially in pathological and frail subjects. Therefore, it seems relevant to analyze the possible existence of cross-effects related to postural control through fatigue effects and training effects induced by acute and chronic exercises, respectively.

### Analysis of Postural Control

Since the aim was to evaluate the possible existence of cross-education related to ipsilateral limb activity on monopedal postural control, only the quantitative evaluation of postural control was taken into account. In studies dealing with this topic, the quantitative analysis of postural control was carried out mainly by measuring the displacement of the center of mass, the center of foot pressure, the body segments and/or the electromyographic (EMG) activities of postural muscles in static (without deformation and displacement of the base of support) and dynamic (deformation and displacement of the base of support) condition. Globally, it turns out that the smaller the displacement of the center of mass, center of foot pressure, and/or the body segments (and/or the weaker the EMG activities), the better the postural control (Paillard and Noe, 2015).

## Cross-Education and Postural Control: Influence of Fatigue

### Available Data

As far as is known, few studies have focused on the transferable effect of the fatigue of the ipsilateral limb musculature on contralateral monopedal postural control. Moreover, the analysis of this topic requires certain considerations beforehand. Since the efficiency of the motor function is essential to the output of the postural system (Paillard, 2017), it is relevant to evaluate the fatigue state of the neuromuscular system (specific) concurrently with that of the postural system (general). In addition, the objective analysis of fatigue effects on contralateral monopedal postural control involves the evaluation of both central and peripheral fatigue by means of techniques (often electrophysiological, such as the twitch interpolation technique and transcranial magnetic stimulation which are used to assess the excitability of different muscle groups) which can make this distinction.

Paillard et al. (2010) first observed that exercises generating central fatigue only i.e., without peripheral fatigue (through a workload including 10 sets of 50 repetitions of 4s-2s- on/off-, each contraction at 10% maximal voluntary contraction – MVC-, i.e., each session >33 min) at the quadriceps femoris level, induced the disturbance of the contralateral monopedal postural control. Moreover, the exercise-induced fatigue of the ipsilateral plantar flexor muscle (using a task of raising the heel as high as possible whilst standing on one leg) would affect plantar flexor strength and dynamic postural control but not static postural control (Son, 2018). This means that the cross-effect was accentuated as the difficulty of the postural task was increased (dynamic vs. static). Besides, the fatiguing exercise had to be relatively long because a short fatigue exercise (i.e., 15 contractions of 4s–16s, each contraction at 30% MVC, i.e., each session = 5 min) did not induce alteration of motor output (muscle strength and EMG activity) and postural control of the contralateral limb (Arora et al., 2015). Based on the results mentioned above, the duration (and probably also the intensity, i.e., intensity-duration interaction) of a fatiguing exercise of the ipsilateral limb is likely to impact monopedal postural control of the contralateral limb. The type of experimental protocol undertaken by Arora et al. (2015) would probably provoke more peripheral fatigue than central fatigue. On the basis of current knowledge, since cross-education depends on central components rather than peripheral components, this result would be logical. Hence, the nature of fatigue (central, peripheral or both) would influence (or not) its disturbing effects of the contralateral monopedal postural control. In the presence of central fatigue alone, contralateral monopedal postural control would be negatively affected while peripheral fatigue alone could be insufficient for disturbing it.

Taking all the results together, one can deduce that exercises of sufficient duration (and probably intensity) of the ipsilateral limb can modify postural control of the contralateral limb if they generate central fatigue.

### Mechanistic Explanations Suggested

The alteration of contralateral monopedal postural control after the completion of a fatiguing exercise of the lower limb could emanate from disturbance of the central command of postural muscles (Paillard, 2012). In fact, this disturbance could arise from the affected activity of the motor units of the ipsilateral muscles. Indeed, changes in medullary reflex impulses of the ipsilateral limb can degrade the drive of the homologous motor units of the contralateral muscles (Rattey et al., 2006) and alter the contralateral motor command (Hortobagyi et al., 2003).

Moreover, Paillard et al. (2010) compared the effects of electro-induced vs. voluntary contractions of quadriceps femoris on cross-over fatigue with the same workload (10% of MVC) on postural control. They reported that the contralateral monopedal postural control was equally disturbed for the two exercises. In fact, the impact of the voluntary exercise and that of the electro-induced exercise would differ between supraspinal and spinal fatigue but overall the effects of fatigue on the contralateral monopedal postural control would be identical. As part of submaximal muscle actions, the voluntary exercise engenders the recruitment of muscle fibers modulated as a function of motor unit size, beginning with the small type I fibers (Henneman and Olson, 1965), whereas the electro-induced exercise of the quadriceps femoris provokes the recruitment of superficial muscle zones near the electrode which display a majority of type II fibers (Lexell et al., 1983). Nevertheless, in cross-over fatigue studies, exercises were carried out and evaluated on the ipsilateral leg only which exclude the opportunity of observing peripheral fatigue in the contralateral leg. Effectively, Paillard et al. (2010) found that the MVC of the contralateral leg was not altered after both fatiguing exercises. It is thus improbable that disturbances in postural control emanating from cross-over fatigue are related to different types of motor units recruited by voluntary or electro-induced muscle actions.

In fact, the voluntary exercise is controlled by central drive while the electro-induced exercise is based on an artificial muscular activation. Voluntary contractions engender changes in drive to the homologous motoneurons (Frazer et al., 2018). Those changes could emanate from inhibitory interhemispheric connections from the motor cortex, receiving projections from the exercised limb, to the homologous motor cortex projecting to the non-exercised limb (Hortobagyi et al., 2003; Todd et al., 2003; Martin and Rattey, 2007; Frazer et al., 2018). Besides, the fusimotor drive to muscle spindles could be altered during the voluntary exercise but not during the electro-induced exercise (Hortobagyi et al., 2003). Hence, it is feasible that the higher cross-over fatigue after a voluntary exercise could stem from alterations in the descending command on the homologous motor units of the contralateral quadriceps femoris. Nevertheless, such alterations should be specifically evaluated to define their role on postural control.

Moreover, physiological data have shown that electro-induced muscle actions corresponding to 10% MVC acidify the cytoplasm and lower the intracellular pH more than voluntary muscle actions within the contracting muscle fibers (Bergstrom and Hultman, 1988; Vanderthommen et al., 2003). These intracellular metabolic changes spill over to the extracellular space and consequently increase the inhibitory effect of type III and IV muscle afferents which involves α-motoneuron inhibition at the spinal level (Rotto and Kaufman, 1988). Reflex inhibition arising from activity of type III and IV muscle afferents in the fatigued muscles could repercut on muscles of the contralateral limb (Martin and Rattey, 2007). In reality, the activity of these afferents could modify the gain of intraspinal neural circuit that may imply the contralateral limb motoneurons through commissural projections. These chemical disturbances would degrade the excitatory effects of the motoneurons of the contralateral muscle more after the electro-induced muscle actions than after the voluntary muscle actions. However, Paillard et al. (2010) found that postural control was not affected differently between the voluntary and electro-induced exercises in’s study, so it would be appropriated to examine the possible inhibitory effects related to acidity on postural control. Kennedy et al. (2015) indeed suggested that cross-over of central fatigue in the lower limb would be not mediated by group III and IV muscle afferents. Furthermore, it would be also relevant to compare the effects of spinal fatigue to those of supraspinal fatigue on the contralateral monopedal postural control.

Globally, both supraspinal and spinal disturbances linked to fatigue are theoretically likely to negatively affect the contralateral monopedal postural control.

### Summary

Fatigue is likely to engender disturbances of contralateral monopedal postural control, particularly if it is of the central nature. From these data concerning the effects of acute exercise, it seems appropriated to analyze the effects of long and intense unilateral exercises repeated regularly (several times a week) i.e., training effects, on contralateral monopedal postural control.

## Cross-Education and Postural Control: Effects of Training

### Available Data

Table 1 describes the results reported by different studies dealing with the effects of training the ipsilateral limb on the contralateral monopedal postural control. This possible cross-education phenomenon was analyzed on the basis of balance training or resistance training (trainings tested by the authors dealing with this topic).

**TABLE 1:** Effects of the ipsilateral limb training on the ipsilateral and/or contralateral monopedal postural control.

	Subjects	Training program		Pre/Post program evaluation	Results/differences (measured variables)
		Exercises/motor tasks	Frequency, duration		
Kadri et al. (2017)*	36 healthy male subjects divided into 3 groups: – 12 VOL – 12 NMES – 12 CON	43 contractions of the quadriceps femoris of the ipsilateral limb at 20% MVC -VOL isometric contractions -NMES electro-induced contractions	3 sessions a week for 8 weeks	– MVC – Static and dynamic monopedal PC	IPSI-MVC: ↑ for VOL and NMES (CON =) CONTRA-MVC: ↑ for VOL and NMES (CON =) IPSI static PC: = for VOL and CON (NMES ↓) IPSI dynamic PC: = for VOL, NMES, CON CONTRA static PC: = for VOL, NMES, CON CONTRA dynamic PC: = for VOL, NMES, CON

Schlenstedt et al. (2017)	51 healthy elderly subjects divided into 2 groups : – 26 TRAIN – 25 CON	Unilateral balance training of the dominant leg during 15 min (6 exercises, each performed 3 times for 30 s and 20 s rest after each repetition)	3 sessions a week for 4 weeks	Static monopedal PC	IPSI-PC: ↑ for TRAIN (CON =) CONTRA-PC: ↑ for TRAIN (CON =)

El-Gohary et al. (2016)	60 healthy subjects divided into 2 groups: – 30 TRAIN – 30 CON	Unilateral proprioceptive training (single leg training on the dominant leg on balance board, minitrampoline, etc.)	3 sessions a week for 8 weeks	– Static monopedal PC – Proprioception repositioning accuracy of the knee joint	CONTRA-PC: ↑ for TRAIN (CON =) Proprioception accuracy: ↑ for TRAIN (CON =)

Hale et al. (2014)	27 subjects with chronic ankle instability – 13 TRAIN – 14 CON	Unilateral balance training (single-legged stance, single-legged hop, wobble board, etc.) on the stable ankle during 30 min	2 sessions a week for 4 weeks	Functional postural tests with stable (IPSI) and unstable (CONTRA) ankle	IPSI-PC: ↑ for TRAIN (CON =) CONTRA-PC: ↑ for TRAIN (CON =)

Oliveira et al. (2013)	23 healthy male subjects divided into2 groups: – 13 TRAIN – 10 CON	Unilateral balance training (single leg stance on the right limb in static condition, on foam pads, dyna discs, wobble boards, etc.) during 25 min	4 sessions a week for 6 weeks	Dynamic monopedal PC EMG onset activity of the lower limb	IPSI-PC: ↑ for TRAIN (CON =) CONTRA-PC: ↑ for TRAIN (CON =) IPSI-EMG: for TRAIN (CON =) CONTRA-EMG: for TRAIN (CON =)

Kim et al. (2011)*	32 healthy subjects divided into 2 groups: – 16 TRAIN – 16 CON	5 sets of 10 concentric isokinetic contractions (unilateral hip exercise) at 60°/s with a rest period of 1–2 min between sets	4 sessions a week for 2 weeks	Static monopedal PC	CONTRA-PC : ↑ for TRAIN (CON =)

Rasool and George (2007)	30 healthy male subjects divided into 2 groups: – 16 TRAIN – 14 CON	Progressive single-leg balance training (simple static balance exercises to more complex and challenging dynamic balance exercises)	5 sessions a week for 4 weeks	Functional postural tests	IPSI-PC: ↑ for TRAIN (CON =) CONTRA-PC: ↑ for TRAIN (CON =)

Osborne et al. (2001)	10 subjects with lateral ankle sprain that occurred more than 6 months but less than 18 months before	Ankle disk training during 15 min	Daily session for 8 weeks	EMG onset activity with injured (IPSI) limb and control (CONTRA) limb (lower limb)	IPSI-EMG: ↑ CONTRA-EMG: ↑

*VOL, voluntary training; NMES, neuromuscular electrical stimulation training; MVC, maximal voluntary contraction; PC, postural control; EMG, electromyography; IPSI, ipsilateral limb; CONTRA, contralateral limb; CON, control subjects; TRAIN, trained subjects; ↑, improvement; =, similar; ↓, regression; ^∗^Kadri et al. (2017)’ resistance exercises were carried out in closed kinetic chain (isometric exercises) while Kim et al. (2011)’ resistance exercises were carried out in (semi)open kinetic chain (isokinetic exercises).*

All the studies carried out on the basis of balance training (in various postural conditions: static, dynamic, unstable, progressive postural difficulty, with or without sensory manipulation) show that ipsilateral and/or contralateral monopedal postural control was improved after periods of 3–5 sessions weekly for 4–8 weeks (Rasool and George, 2007; Oliveira et al., 2013; Hale et al., 2014; El-Gohary et al., 2016; Schlenstedt et al., 2017). By contrast, the effects of resistance training of the ipsilateral limb on the contralateral monopedal postural control are much less clear since Kim et al. (2011) reported that contralateral monopedal postural control was improved after 4 sessions weekly for 2 weeks while Kadri et al. (2017) concluded that contralateral monopedal postural control was not improved after 3 sessions weekly for 8 weeks with either voluntary contractions or electro-induced contractions.

Moreover, Oliveira et al. (2013) and Osborne et al. (2001) reported muscle onset latency decreases (i.e., neuromuscular responses to postural perturbations) in muscles regulating monopedal postural control (e.g., anterior tibialis muscle) after unilateral balance trainings (4 sessions weekly for 6 weeks or daily session for 8 weeks, respectively) for the contralateral limb which suggests a possible proprioceptive cross-training effect in both healthy and pathological subjects, respectively. El-Gohary et al. (2016) also reported that proprioceptive training including mainly balance exercises on a single leg in different sensory and environmental conditions three times per week over a period of 8 weeks induced cross-training effects on proprioception repositioning accuracy of the knee joint (decrease in the repositioning error and better joint position sense) and on postural control (better postural control) of the untrained leg in healthy subjects.

Evidently, the unilateral balance training generates cross-education of contralateral monopedal postural control. The results of studies carried out on the basis of resistance training deserve to be completed in order to formally respond to the question raised.

### Mechanistic Explanations Suggested

Improvement in monopedal postural control of the contralateral limb after balance training can be ascribed to neural adaptations (Rasool and George, 2007; Oliveira et al., 2013; Hale et al., 2014; El-Gohary et al., 2016; Schlenstedt et al., 2017). The studies that have evaluated the effects of resistance training are not as clear and unanimous (Kim et al., 2011; Kadri et al., 2017). Balance training being specific to the evaluation task, whereas resistance training is not, it would be logical to assume that possible adaptations would not have the same origins. Thus, the neural adaptations accompanying the cross-education phenomenon can be illustrated through two assumptions. The first one would mean that resistance training engenders changes in the organization of motor pathways projecting to the contralateral homologous muscle, while the second one involves resistance training as a form of motor learning (Carroll et al., 2006; Schlenstedt et al., 2017). The control and execution of postural tasks of the trained limb could be improved through changes in circuits that may be accessible by the opposite hemisphere. These changes would reflect potential motor learning in postural tasks.

#### Regarding Balance Training

Relevant balance training includes varied and progressive postural tasks ranging from simple static balance exercises to more complex and challenging dynamic balance exercises (e.g., with eyes open and closed, on solid and soft supports, with and without additional motor and cognitive tasks). In this way, the activity of the ipsilateral monopedal limb involves and stimulates the processing and integration of visual and vestibular information, which improving with training, benefit postural control in the contralateral monopedal condition. With regard to the processing and integration of proprioceptive information, the situation is different since the specific myotendinous and articular chains of the contralateral limb are not stimulated. From the point of view of proprioceptive information and its integration as well as motor command, cross-education related to postural control would mainly result from central adaptations.

Balance exercises augment the attention paid to proprioceptive cues by the brain, first at the conscious level early in training, then later, after further training, at the autonomous level (Rasool and George, 2007). This could occur as a central processing adaptation rather than just a peripheral cue sensitization. Moreover, Oliveira et al. (2013) suggested that muscular onset postural adaptations from the trained limb were also reported in the untrained limb, accompanied by reduced EMG activity (time to peak) which may have been acquired by cortical interconnections that transfer adaptations between limbs. In addition, the neural drive from motor cortex to motor muscles (cortical voluntary activation) would be enhanced after unilateral training and mediated by cross-spinal pathways (Farthing et al., 2011). Hence, the motor output of the contralateral homologous muscle could be improved in monopedal postural tasks. The improvement of contralateral monopedal postural control may be a result of better coordination between the hip abductor and adductor muscles after isokinetic training (Kim et al., 2011). In postural control involving coactivation of agonist and antagonist muscles, after training the degree of coactivation of antagonists can decrease while that of agonists can increase in the untrained leg (Oliveira et al., 2013). This improved agonist-antagonist coordination can facilitate the contralateral monopedal postural control.

#### Regarding Resistance Training

Unilateral resistance training can improve contralateral postural control (Kim et al., 2011) but it was also shown that the increase in muscle strength was not sufficient to improve contralateral monopedal postural control (Kadri et al., 2017). Kim et al. (2011) studied resistance exercises completed in dynamic condition (isokinetic contractions at 60°/s) while Kadri et al.’s (2017) studied resistance exercises performed in static condition (isometric and electro-induced contractions). In fact, Kim et al. (2011)’ exercises was carried out in (semi)open kinetic chain (i.e., the distal extremity was quasi-free and subjected to resistance) while Kadri et al. (2017)’ exercises were completed in closed kinetic chain (i.e., the distal extremity was fixed). This means that Kim et al. (2011)’ exercises engendered joint mobilizations while Kadri et al. (2017)’ exercises did not. In addition, the extent of the muscle chains solicited was greater for Kim et al. (2011)’ exercises than for Kadri et al. (2017)’ exercises (i.e., extensor and flexor hip muscles as well as abductor and adductor hip muscles vs. only knee extensor muscles). Hence, only training programs using dynamic and multi-joint exercises would be likely to improve the monopedal postural control in healthy subjects (Kadri et al., 2017). Moreover, the protocol of Kim et al. (2011) included maximal contractions while that of Kadri et al. (2017) included submaximal contractions (20% MVC). Maximal/intense workloads could be more effective than submaximal/light workloads to induce positive neural adaptations related to cross-education as part of postural control. Finally, the kinetic conditions (relative conditions to movement) of resistance exercises are different from specific movements relative to the postural evaluation task, so it would be logical that the adaptations differ from those of balance exercises that are specific to the postural evaluation. Indeed, the postural adaptations induced are specific to the context in which the exercise is practiced (Paillard, 2017). This author concluded that they are so specific that there would be no or only a very slight effect of transfer to non-experienced motor tasks. Hence, one can theoretically assume that it is more difficult to improve postural control after resistance training than after balance training especially on the contralateral limb.

### Summary

**FIGURE 1:**
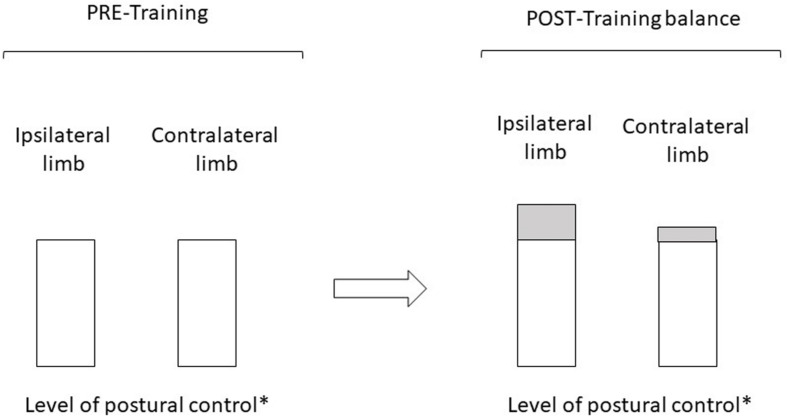
The effects of unilateral balance training on the contralateral monopedal postural control are beneficial. Evidence suggests that the postural gains induced by unilateral training balance – i.e., POST-training condition on the figure- (represented by gray rectangles) are nevertheless greater for the ipsilateral limb (trained limb) than for the contralateral limb (untrained limb). ^∗^The level of postural control is inversely proportional to kinetic, kinematic and EMG values measured. In fact, the smaller the displacement of the centre of mass, centre of foot pressure, and/or the body segments (and/or the weaker the EMG activities), the better the postural control (Paillard and Noe, 2015).

Cross-education related to postural control would emanate from spinal and supra-spinal adaptations but would require optimal training conditions (notably the nature and specificity of movement as well as the physiological and/or psychological involvement of subjects). Unilateral training produces cross-education related to postural control especially when it includes balance exercises even though the evidence suggests that the gains postural are greater for the ipsilateral limb than for the contralateral limb (Figure 1). However, this cross-education phenomenon remains to be tested when it includes resistance exercises.

## Study Limitations and Future Perspective

The number of studies have dealt with the effects of the ipsilateral limb activity on monopedal postural control of the contralateral limb is currently too limited. Only a narrative review was possible on the basis of the data currently available on the topic. Future work is needed to determine conclusively whether or not there are cross-education effects with respect to postural control. As part of the effects of balance training, all the studies carried out have shown that ipsilateral limb activity is effective in improving monopedal postural control of the contralateral limb. Nevertheless, the next steps would merit first confirming these previous findings, and then exploring the different postural contexts (e.g., static or dynamic postural tasks, combined motor tasks, combined postural and cognitive tasks) in which the cross-education effects would be greatest. As part of the effects of resistance training, the situation is different because, on the one hand, too few studies have been carried out on this topic, on the other hand, the results of these studies diverge considerably. Thus, further work on this topic is awaited in order to know with certainty whether the ipsilateral leg resistance training is beneficial to the contralateral monopedal postural control.

## Conclusion

The findings show that cross-over fatigue is able to disturb postural control. The contralateral monopedal postural control is impaired, similarly, after fatiguing electro-induced and voluntary contractions. These findings may have useful applications in the therapeutic framework. Effectively, the sessions of rehabilitation following surgery of a lower limb often require the completion of electo-induced and/or voluntary exercises. The deleterious consequences of cross-over fatigue on postural control should be taken into account by therapists in the design of their therapeutic programs.

Further studies are needed to confirm (or invalidate) these results, since few studies have been carried out on this topic. However, it seems that cross-education is favorable to postural control particularly when unilateral training includes balance exercises, but this remains to be confirmed when resistance exercises are included. Therapists and sport trainers could exploit this new knowledge in their professional practice to enhance their preventive and therapeutic strategies for patients and frail/older subjects and to improve their interventions and to refine their training programs for athletes. Therapeutic strategies and (re)training strategies (after rehabilitation program) taking into account this new knowledge could enhance (or avoid losing) the monopedal postural abilities of the initially affected limb and limit its risk of new injury (e.g., from a fall) in patients and athletes. Balance/proprioceptive training programs are effective and feasible and deserves to be systematically incorporate into functional rehabilitation, fall prevention, and sport training programs.

## Author Contributions

The author confirms being the sole contributor of this work and has approved it for publication.

## Conflict of Interest

The author declares that the research was conducted in the absence of any commercial or financial relationships that could be construed as a potential conflict of interest.
